# Transoesophageal echocardiography and extracorporeal membrane oxygenation: fancy for enthusiasts or indispensable tool?

**DOI:** 10.1186/cc13324

**Published:** 2014-03-17

**Authors:** G Auzinger, R Loveridge, C Willars, T Best, V Kakar, T Hurst, A Vercueil

**Affiliations:** 1 King's College Hospital London, UK

## Introduction

Echocardiography is commonly used during both venoarterial (V-A) and venovenous extracorporeal membrane oxygenation (ECMO). In many circumstances, transoesophageal echocardiography (TOE) is the preferred monitoring tool. It can aid in cannula positioning, especially during double-lumen cannula placement for V-V ECMO, weaning of V-A ECMO and diagnose causes of high-access pressures and circuit flow problems. We use TOE as our preferred monitoring equipment before, during and after establishing ECMO. We sought to investigate how often information gained from TOE imaging had a major impact on management decisions.

## Methods

A single-centre observational study at a tertiary referral institution. All patients supported with V-A or V-V ECMO during an 18-month period were included. Routine procedures such as wire position checks during cannulation or information gained to assist weaning from V-A support were not included.

## Results

Twenty patients were supported with either V-A (all peripheral) or V-V ECMO during the observation period. In 12 patients (60%) TOE was instrumental in diagnosing potentially fatal complications or altered clinical management. In three patients on V-A support, afterload reduction and modulation of inotropic support was necessary due to extensive spontaneous echo contrast formation in the left ventricle and stagnant pulmonary blood flow; two out of these three patients, immediately after establishing support, required intra-aortic balloon counterpulsation to reverse clot formation around the aortic valve and root. Further diagnosis were Avalon cannula misplacement in the blind end of a piggyback cava following liver transplant, collapsing right atrial free wall with 'sucking down' into the access cannula (*n *= 3), pericardial clot formation and tamponade (*n *= 1), persistent left superior vena cava making planned cannula placement into the right atrium impossible or potentially fatal, diagnosis of patent foraminae ovale providing left atrial decompression and appropriate drainage cannula positioning during liver transplantation on ECMO.

## Conclusion

In our practice, TOE is an indispensable tool for safe ECMO practice, both during V-A and V-V support.

